# Development of the concept of prenatal care Application of the hybrid model of Schwartz-Barcott and Kim

**DOI:** 10.15649/cuidarte.4387

**Published:** 2025-07-10

**Authors:** Javier Alonso Bula-Romero, Adier Andrés Conde-Flórez, Aura María González-Lara, Álvaro Antonio Sánchez-Caraballo, Gustavo Edgardo Jiménez-Hernández

**Affiliations:** 1 Universidad de Córdoba, Montería, Colombia. E-mail: javierbula@correo.unicordoba.educ.co Universidad de Córdoba Montería Colombia javierbula@correo.unicordoba.educ.co; 2 Universidad de Córdoba, Montería, Colombia. E-mail: acondeflorez@correo.unicordoba.edu.co Universidad de Córdoba Montería Colombia acondeflorez@correo.unicordoba.edu.co; 3 Universidad de Córdoba, Montería, Colombia. E-mail: agonzalezlara@correo.unicordoba.edu.co Universidad de Córdoba Montería Colombia agonzalezlara@correo.unicordoba.edu.co; 4 Universidad de Córdoba, Montería, Colombia. E-mail: aasanchez@correo.unicordoba.edu.co Universidad de Córdoba Montería Colombia aasanchez@correo.unicordoba.edu.co; 5 Universidad de Córdoba, Montería, Colombia. E-mail: gustavojimenezh@correo.unicordoba.edu.co Universidad de Córdoba Montería Colombia gustavojimenezh@correo.unicordoba.edu.co

**Keywords:** Prenatal Care, Maternal Health, Maternal Welfare, Maternal and Child Health, Maternal-Child Nursing, Atención Prenatal, Salud Materna, Bienestar Materno, Salud Materno Infantil, Enfermería Materno Infantil, Atenção Pré-Nata, Saúde Materna, Bem-Estar Materno, Saúde Materno-Infantil, Enfermagem Materno-Infantil

## Abstract

**Introduction:**

Prenatal care is essential for maternal and neonatal health. Nursing professionals play a key role in providing comprehensive care.

**Objective:**

To analyze the concept of prenatal caring in the context of maternal-perinatal care from the perspective of nursing professionals and pregnant women.

**Materials and Methods:**

The concept was developed using the hybrid model by Schwartz-Barcott and Kim, which comprises theoretical, fieldwork, and analytical phases. The first step included a review of 23 articles published between 2012 and 2022, sourced from various databases. Then, five interviews were conducted with low-risk pregnant women and five with nursing professionals, followed by inductive content analysis using ATLAS Ti® software. Finally, a general definition was formulated.

**Results:**

Prenatal caring is defined as a humanized interaction between the pregnant woman, her family, and nursing professionals, grounded in kindness, respect, and commitment. It requires empathy, attentiveness to individual needs, and a focus that goes beyond technical procedures to emphasize holistic well-being.

**Discussion:**

A distinction is drawn between prenatal care and prenatal caring, the latter being understood as a woman- and family-centered process. Operational and time constraints hinder the delivery of this care, highlighting the need to strengthen nursing leadership and foster care models that prioritize maternal and infant well-being.

**Conclusion:**

Prenatal caring in nursing addresses the emotional and relational needs of pregnant women and their families. It enhances adherence to prenatal checkups, contributes to the reduction of mortality, and supports preparation for childbirth. It reaffirms an approach centered on the woman, her unborn child, her partner, and the family.

## Resumen

**Introducción:**

El cuidado prenatal es esencial para la salud materna y neonatal. El profesional de enfermería juega un papel clave al brindar cuidado integral.

**Objetivo:**

Analizar el concepto de cuidado prenatal en el contexto de la atención materno-perinatal desde la perspectiva de los profesionales de enfermería y las gestantes.

**Materiales y métodos:**

El desarrollo del concepto se llevó a cabo utilizando el modelo híbrido de Schwartz-Barcott y Kim, que incorporó las fases teóricas, de campo y de análisis final. El primer paso fue una revisión de 23 artículos disponibles en diferentes bases de datos publicados entre 2012 y 2022. Luego, se realizaron 5 entrevistas a profesionales de enfermería gestantes de bajo riesgo, seguidas de análisis de contenido inductivo, con apoyo del software ATLAS Ti®. Por último, se realizó una definición general.

**Resultados:**

El cuidado prenatal es una interacción humanizada entre la gestante, su familia y los profesionales de enfermería, basada en amabilidad, respeto y compromiso. Requiere empatía, atención a necesidades y trascendencia de lo técnico, enfocándose en el bienestar integral.

**Discusión:**

Se diferencia entre atención y cuidado prenatal, resaltando este último como un proceso centrado en la mujer y su familia. Las limitaciones operativas y de tiempo afectan la asistencia, evidenciando la necesidad de fortalecer el liderazgo enfermero y promover modelos integrales que prioricen el bienestar materno-infantil.

**Conclusiones:**

El cuidado prenatal de enfermería comprende las necesidades emocionales de la mujer gestante y su familia. Deriva en adherencia al control prenatal, menor mortalidad y preparación para el parto. Reivindica el enfoque centrado en la mujer, su hijo, compañero y familia.

## Resumo

**Introdução:**

O cuidado pré-natal é essencial para a saúde materna e neonatal. O profissional de enfermagem desempenha um papel fundamental ao oferecer cuidado integral.

**Objetivo:**

Analisar o conceito de cuidado pré-natal no contexto da atenção materno-perinatal sob a perspectiva dos profissionais de enfermagem e das gestantes.

**Materiais e métodos:**

O desenvolvimento do conceito foi realizado utilizando o modelo híbrido de Schwartz-Barcott e Kim, que incorporou as fases teórica, de campo e de análise final. O primeiro passo foi uma revisão de 23 artigos disponíveis em diferentes bases de dados, publicados entre 2012 e 2022. Em seguida, foram realizadas 5 entrevistas com profissionais de enfermagem gestantes de baixo risco, seguidas de análise de conteúdo indutiva, com o apoio do software Atlas Ti®. Por fim, foi elaborada uma definição geral.

**Resultados:**

O cuidado pré-natal é uma interação humanizada entre a gestante, sua família e os profissionais de enfermagem, baseada em gentileza, respeito e compromisso. Requer empatia, atenção às necessidades e transcendência do aspecto técnico, focando no bem-estar integral.

**Discussão:**

É feita uma distinção entre assistência e atenção pré-natal, destacando esta última como um processo centrado na mulher e sua família. Restrições operacionais e de tempo impactam a prestação de cuidados, destacando a necessidade de fortalecer a liderança de enfermagem e promover modelos abrangentes que priorizem o bem-estar materno e infantil.

**Conclusões:**

O cuidado de enfermagem pré-natal contempla as necessidades emocionais da gestante e sua família. Isso resulta na adesão ao pré-natal, menor mortalidade e preparação para o parto. Ela afirma que a abordagem é focada na mulher, no filho, no parceiro e na família.

## Introduction

Prenatal care is a cornerstone of maternal and neonatal health promotion, and its quality directly influences perinatal outcomes^1^. Within this context, nursing plays a pivotal role, as nurses are often at the forefront of care, providing a range of services that include education, emotional support, complication management, and care coordination^2^. However, the concepts of “prenatal care” and “prenatal caring” have been defined inconsistently across the literature, revealing the need for a conceptual analysis to explain its scope, characteristics, and essential dimensions. 

For years, prenatal care has been regarded as a series of medical interventions aimed at protecting the health of the mother and fetus during pregnancy^3^. However, in recent decades, there has been a growing recognition that prenatal caring extends beyond biomedical interventions to encompass psychosocial and educational components that are fundamental to the overall health of the pregnant woman^4^. In their role as health promoters, nurses have a unique opportunity to provide holistic prenatal care that addresses the multidimensional health needs of pregnant women^5^. 

Analysis of the components of “prenatal caring” is equally important for improvement as it will lead to greater clarity and consistency, which would undoubtedly enhance good clinical practices, professional education, and research related to nursing practice in the community. Conceptualization is critical to developing concepts, measurement tools, and effective interventions^6^,^7^. However, despite the importance of defining this concept, its implementation is not straightforward, particularly when addressing nursing and public health issues. The lack of a clear working definition of “public health nursing” further fragments and complicates the effective provision of prenatal care given the absence of a well-defined scope and standards^8^. 

The existing literature on prenatal nursing care has produced various themes and models that seek to illustrate the care provided during pregnancy. Some highlight emotional and psychological support as central to prenatal caring nursing^6^,^9^, while others emphasize the need for educational strategies to promote self-care and informed decision-making among pregnant women^9^. While these contributions are valuable, certain aspects of prenatal caring in nursing practice remain unclear, particularly regarding how nurses understand and apply this reasoning across different cultural and care contexts. 

According to Ruiz and Muñoz^10^, prenatal caring involves an encounter between the healthcare team and the pregnant woman arising from a need for caring that emerges during the gestational process^10^. This encounter represents a specific form of intersubjective relationship in which the individuals involved (health professionals, the pregnant woman, and her family) share experiences that foster the recognition of real caring needs extending beyond the mere performance of activities, procedures, and interventions aimed at the early detection of pregnancy-related complications^11^. 

Prenatal care, by contrast, is understood as the set of activities, procedures, and interventions aimed at improving maternal health and promoting fetal development during the prenatal period^12^. In this sense, prenatal care encompasses the technical and operational aspects of the healthcare process for the pregnant woman. However, the term is often confused with the concept of prenatal caring, as many healthcare professionals tend to equate "taking care" with "caring for." This situation creates a fine line between the two concepts that require clarification^13^. Clarifying the concept of prenatal caring is fundamental for nurses to use it accurately and to advance new theoretical developments in this area of disciplinary knowledge. 

Over the past four decades, theoretical contributions to the nursing discipline have expanded, as has the number of methods for generating and analyzing nursing concepts^14^-^17^. From this perspective, the study of concepts is considered essential for two main reasons: first, concepts are used to construct and develop new theories^18^; and second, they can be applied in practice to improve it^19^. 

Using the hybrid model developed by Schwartz-Barcott and Kim^20^, an appropriate conceptual analysis can be achieved, one that is solidly integrated at both theoretical and empirical levels. This model has proven particularly useful in nursing practice, where the concept under analysis is complex in nature and requires both an exhaustive review of the literature and the exploration of the lived experiences of nurses working in the field of prenatal nursing. Applying this approach to the analysis of the concept of “prenatal caring” should facilitate not only more accurate and context-specific definitions but also an understanding of how nurses are prepared to effectively address challenges and seize opportunities to deliver high-quality prenatal care. 

For this reason, this research aims to conduct a detailed analysis of the concept of “prenatal caring” using the hybrid model approach developed by Schwartz-Barcott and Kim. The analysis seeks to clarify the core elements of the concept, identify barriers and facilitators in clinical practice, and put forward proposals to strengthen nursing education and practice within the prenatal care setting. It is hoped that in doing so, a foundation will be laid for more consistent and effective practices that reflect the best available evidence and the specific needs of pregnant women. 

## Materials and Methods

**Study design**

This study was conducted using the hybrid model of concept analysis proposed by Schwartz-Barcott and Kim^20^, which consists of three phases: the theoretical phase, involving an extensive review of the literature; the fieldwork phase, based on the collection and analysis of qualitative data; and the analytical phase, in which data from the two previous phases were analyzed, compared, and integrated to produce a more robust definition of the concept. 

The hybrid model developed by Schwartz-Barcott and Kim^20^ is a valuable methodological tool for clarifying and developing complex concepts in nursing practice. This approach integrates theoretical and empirical elements, enabling a comprehensive exploration of the concept of “prenatal caring.” The methodology unfolds across three interrelated phases: the theoretical phase, the fieldwork phase, and the analytical phase. Each phase plays a critical role in formulating a precise definition of the phenomenon under study. 

**Theoretical phase**

To initiate this study, we conducted searches in PubMed, BVS, Scopus, ProQuest, and ScienceDirect, selecting articles relevant to our study. Inclusion criteria comprised original research articles, literature reviews, theoretical reflections, and book chapters published in the specified databases using the following keywords: Prenatal Care, Maternal and Child Health, Maternal-Child Nursing, Maternal-Child Health Services, Perinatal Care, and Maternal Health. To broaden or narrow the search strategy, Boolean operators (OR and AND) were used in combination, and publications in English, Portuguese, and Spanish were considered. 

**Figure 1:**
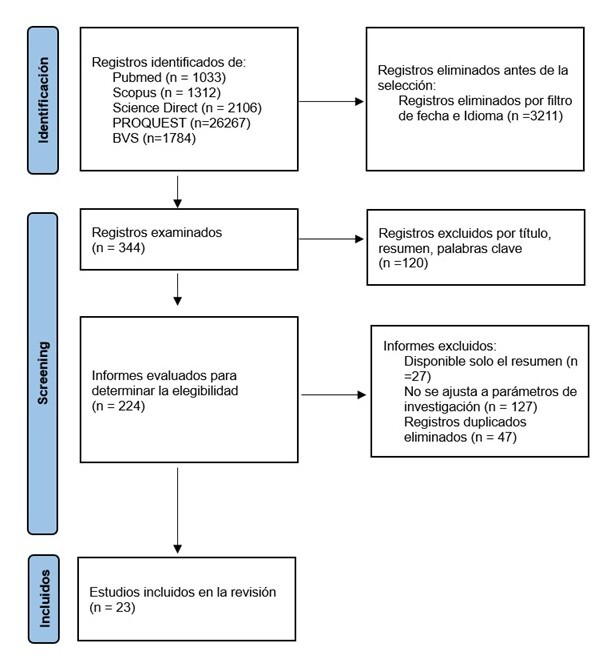
Identification of studies via databases and registers

A critical appraisal of the selected articles was conducted, which is useful for the analysis and synthesis of the available empirical information. The full text of each eligible article was downloaded and evaluated according to the inclusion criteria: open-access articles in English, Portuguese, or Spanish, published between 2012 and 2022. Exclusion criteria comprised duplicates, gray literature, articles not published in journals indexed within the selected databases for the integrative review, and those without an available abstract. All discrepancies in the final inclusion of articles were discussed and resolved with the mediation of a third reviewer, who did not participate in the initial selection process (Figure 1). 

Data loss was controlled by categorizing the articles; to this end, the researchers designed a matrix in Microsoft Excel to compile and systematize the information. Classification bias was mitigated by adhering to the methodological rigor characteristic of systematic reviews, strictly following the procedures outlined in the methodological design. 

**Fieldwork Phase**

In the second phase, qualitative data were collected through semi-structured interviews. To ensure maximum variation^21^, pregnant women representing a range of age groups, socioeconomic strata, and educational levels were interviewed. Inclusion criteria comprised physical ability to participate in the study, being 18 years of age or older, literacy and verbal communication skills, willingness to participate and consent to the recording of the interview, and the ability to distinguish nursing professionals from other healthcare providers involved in prenatal care. 

The selection of participants met the criteria of variability and representativeness. Variability in this research was achieved through the representativeness of the study participants who worked in prenatal care services in the city of Monteria, department of Cordoba. According to Castillo^22^, representativeness, refers to participants having direct experience with the phenomenon under study. For this research, nurses working in maternal-perinatal outpatient healthcare settings were selected. 

Convenience sampling was used to select the nursing professionals, and the group of participants was assembled using a snowball strategy^23^. The inclusion criteria for nurses were as follows: being a nurse with a minimum of five years of experience in the prenatal care area, with or without postgraduate education in fields related to maternal-perinatal care, currently working in outpatient care settings in the city of Monteria, and actively performing prenatal checkups. 

Once approval was obtained from the health institution, along with written informed consent and permission to record the interviews, interviews with pregnant women were conducted separately from those with nurses in private areas to minimize interference. To support participant comfort, their characteristics and the research context were described, and the data collection and analysis process was detailed step-by-step. Regarding auditability, the principal researchers manually performed the initial data analysis, organizing the information using a matrix designed in Microsoft Excel. The data were subsequently audited and validated by the advisor of the final graduation project, with the support of ATLAS.ti® software, version 22 (license No. L-EC1-811). 

Credibility was established through the training of researchers in qualitative interviewing techniques. The first two interviews, conducted as part of the exploratory study, were audited by the advisor of the final graduation project, who provided recommendations for refining the final interview guide. Auditability was ensured through the verbatim transcription of all interviews. 

In the final step, to validate the study's results, the participating nurses were invited to take part in a focus group. This session was conducted online via Google Meet with participants who had been previously contacted and had agreed to participate. The meeting lasted approximately sixty minutes and focused on reflections regarding nursing professional practice in relation to prenatal care. The articles selected for this study are stored in, and accessible through, Mendeley Data^24^. 

**Ethical considerations**

The study was conducted in accordance with the ethical principles outlined in the Declaration of Helsinki and Resolution 8430 of 1993 of the Colombian Ministry of Health^25^. It was classified as a risk-free research study, with all efforts directed toward safeguarding participants’ rights. The research was reviewed and endorsed by the Accreditation and Curriculum Committee of the Nursing Program at the Universidad de Córdoba. It was submitted for evaluation by academic peers, who assessed the ethical and methodological aspects of the study. Prior to data collection, written informed consent was obtained, the purpose of the study was explained, and participants were informed that their participation was voluntary. A private space (consulting room) was provided by the participating institutions to ensure the privacy and comfort of the study participants. Anonymity was maintained, and data confidentiality was strictly upheld. The ethical principles of beneficence and nonmaleficence were respected to ensure that participation in the interviews did not cause discomfort or emotional harm. Findings were reported with full disclosure of the information and were used exclusively for academic and scientific purposes. 

## Results

The initial search yielded 32,502 articles, followed by a pre-selection based on title, abstract, and keywords, which resulted in 344 articles. Of these, 224 articles were excluded for not being related to the purpose of the study. Finally, 23 articles addressing the study phenomenon were selected. The majority of the selected studies, 65.21% (n = 14), employed a qualitative approach^26^-^40^, utilizing exploratory designs, semi-structured interviews, participant observation, focus groups, content analysis, or phenomenological approaches. In turn, 30.43% (n = 7) corresponded to quantitative studies, including descriptive studies, systematic reviews, and quasi-experimental studies^3^,^9^,^41^-^45^. One study with a mixed-methods approach was also identified^45^. 

Of the studies analyzed (n = 19), 82.60% were conducted in Latin America^27^-^41^,^43^-^45^,^46^, while Africa^9^,^26^, Europe^42^, and Asia^3^ contributed two articles each (9.26%) and one article (4.34%), respectively. Within Latin America, Brazil had the highest contribution, representing 47.82% (n = 11) of the studies, followed by Colombia with five articles (21.73%). 

The term cuidado prenatal in Spanish is a two-part structure composed of the participle of the verb "to care" and an adverb that qualifies the verb. Etymologically, the word cuidado derives from the Latin cōgitātus, which conveys the ideas of reflection, thought, and attention directed toward something. It is the participle form of cōgitāre, also meaning reflection directed toward something. Notably, the word structure includes the prefix co-, which implies joint or collective action^47^. 

Similarly, the term prenatal in both Spanish and English derives from Latin and refers to everything that occurs before birth. Its components include the prefix pre-, meaning "before," the root nātus, meaning "born," and the suffix -al, meaning "related to." Thus, based on its Latin origins, it can be inferred that the term cuidado prenatal refers to an action that arises from a reflective process directed toward the well-being of an individual who is about to be born^48^. 

**Low-risk prenatal nursing care**

Low-risk prenatal nursing care is influenced by the biomedical model^28^,^30^,^38^,^43^,^45^. This model of care has focused on identifying risk factors that may alter or compromise the normal course of pregnancy. Within this framework, nurses are responsible for leading prenatal care processes^27^,^28^,^30^,^35^,^36^ and coordinating care for pregnant women in collaboration with other members of the multidisciplinary team to provide comprehensive, integrated, and continuous prenatal care^26^,^29^,^45^ (Table 1). 

Low-risk prenatal care provided by nurses is grounded in evidence-based interventions^3^,^32^,^43^. It is characterized by being periodic, sequential^26^,^29^,^45^, and coordinated with other disciplines^26^,^31^,^32^. In this context, nursing professionals are required to possess cognitive and relational competencies, along with skills and abilities for the care management of pregnant women and their unborn children during the prenatal period^26^,^29^,^30^,^40^. Prenatal care should be humanized and centered on the woman and her family^26^,^31^,^32^,^35^,^36^,^45^. 

**Low-risk prenatal nursing consultation**

Prenatal care for low-risk pregnant women is implemented through prenatal checkup consultations^27^,^28^,^30^,^35^,^36^. During this type of consultation, the pregnant woman undergoes physical and psychosocial assessments^26^,^27^,^29^,^30^,^35^, routine care activities are performed^26^,^27^,^29^,^30^,^43^, prenatal counseling and education are provided^27^-^29^,^35^,^40^,^46^, and ongoing follow-up is ensured throughout the prenatal period^26^,^29^,^30^,^43^,^45^. 

**Implicit attributes of low-risk prenatal caring nursing**

In describing the attributes of prenatal caring provided by nurses, certain values that characterize their practice are identified. These values include respect for women, a sense of responsibility, and kindness in prenatal caring (Table 2). 

**Table 1:** References of articles containing antecedents of the concept of prenatal caring

Indicators. Low-risk prenatal nursing care
Prenatal care is influenced by the biomedical model (risk-based approach)^28^,^30^,^38^,^43^,^45^
Prenatal care is evidence-based^3^,^32^,^43^
It is periodic, sequential, comprehensive, integrated, and continuous^26^,^29^,^45^
It is multidisciplinary^26^,^31^,^32^
Nurses are required to demonstrate cognitive, relational, technical, and instrumental skills, as well as competencies in care management to deliver prenatal care^26^,^29^,^30^,^40^.
It is humanistic and centered on the pregnant woman and her family^26^,^31^,^32^,^35^,^36^,^45^.
Indicators. Activities during prenatal nursing consultation
Interaction occurs between the nurse and the pregnant woman during prenatal consultation^27^,^28^,^30^,^35^,^36^.
Knowledge, experiences, and care practices are shared^27^,^28^,^31^,^35^,^40^.
Physical and psychosocial assessments are conducted^26^,^27^,^29^,^30^,^35^.
Nurses perform routine prenatal care activities during prenatal consultation^26^,^27^,^29^,^30^,^43^.
Prenatal counseling and education are provided^27^-^29^,^35^,^40^,^46^.
Pregnant women are monitored throughout the prenatal period^26^,^29^,^30^,^43^,^45^.

Source: Data analysis based on the literature review.

**Table 2:** References of articles containing the attributes of the concept of prenatal caring

Indicators Values characterizing nursing professionals providing prenatal caring.
Respect ^26^,^32^,^35^,^36^,^38^.
Responsibility^26^,^29^,^30^,^37^,^40^.
Kindness^27^,^35^,^36^,^38^.
Indicators Implicit attributes of low-risk prenatal caring nursing
Emphathy^27^,^32^,^35^,^36^,^38^.
Welcoming attitude^26^,^27^,^35^,^36^.
Support^3^,^26^,^31^,^32^,^39^.
Commitment^26^,^29^,^30^,^37^,^40^.
Concern^26^,^29^,^37^,^40^.
Active listening^27^,^28^,^35^,^36^.
Sensitivity^27^,^32^,^35^,^36^,^38^ .

Source: Data analysis based on the literature review.

It is evident that prenatal caring has a different connotation than prenatal care. The literature reviewed highlights that empathy^26^,^27^,^35^,^36^,^38^; welcoming the pregnant woman and her family^26^,^27^,^35^,^36^; providing support during the gestational process^3^,^26^,^31^,^32^,^39^; caring about them^26^,^29^,^37^,^40^; practicing active listening^27^,^28^,^35^,^36^; demonstrating sensitivity to issues related to the experience of motherhood; and fostering commitment to the caring of the pregnant woman and her unborn child^27^,^32^,^35^,^36^,^38^ are attributes or distinguishing features of prenatal caring. 

**Consequences of low-risk prenatal caring performed by nursing for the mother-child dyad**

Among the consequences of low-risk prenatal caring provided by nurses for the mother-child dyad, the literature review indicates that nursing professionals contribute to empowering women throughout the gestational process^29^,^31^,^32^,^35^,^40^ by promoting self-care^36^ and fostering a positive pregnancy experience^3^,^26^,^27^,^32^,^35^,^38^. This interaction supports the physical and psychological preparation of the woman for childbirth and puerperium^3^,^27^,^28^,^31^,^32^,^40^ and strengthens the mother-child bond during the prenatal period^26^,^31^,^34^,^35^. It also enhances the woman's confidence as she transitions into motherhood^27^,^31^,^32^,^35^,^40^. These elements contribute to the prevention of maternal and perinatal complications and lead to improved maternal and perinatal health outcomes^27^,^28^,^35^,^36^,^46^, including increased satisfaction with prenatal caring^9^,^26^,^29^,^30^,^45^, greater adherence to prenatal checkups, and reductions in maternal and perinatal mortality^9^,^42^,^45^ (Table 3). 

**Table 3:** References of articles containing the consequences of the concept of prenatal caring

Indicators For the mother
Empowerment of women in the gestational process and management of their own care ^29^,^31^,^32^,^35^,^40^
Facilitation of a positive pregnancy experience^3^,^26^,^27^,^32^,^35^,^38^
Physical and psychological preparation for childbirth and puerperium^3^,^27^,^28^,^31^,^32^,^40^.
Strengthening of the mother-child bond during the prenatal period^26^,^31^,^34^,^35^.
Improvement of women’s confidence in transitioning to motherhood^27^,^31^,^32^,^35^,^40^
Indicators For the child to be born
Reduction of perinatal mortality^9^,^42^,^45^
For the maternal-perinatal health indicators
Satisfaction with prenatal caring nursing ^27^,^28^,^35^,^36^,^46^
Adherence to prenatal nursing checkups^9^,^26^,^29^,^30^,^45^
Reduction in maternal and perinatal mortality^9^,^42^,^45^

Source: Data analysis based on the literature review.

**Results of the fieldwork phase**

Ten participants were interviewed, including five nursing professionals and five pregnant women at low obstetric risk. The interviews were conducted over a three-month period, with durations ranging from 30 to 60 minutes. ATLAS.ti software, version 22, was used for data analysis. The interviews were transcribed verbatim and analyzed. Theoretical saturation was reached when the categories and subcategories identified in the analysis were sufficiently explained. 

The content analysis technique was applied through the following steps:

1. The transcribed texts were read and reread, highlighting fragments that reflected the antecedents, attributes, and consequences of the concept of prenatal caring.

2. Relevant excerpts from the interviews were coded using conceptual memos and quotations, with a focus on identifying elements related to the antecedents, attributes, and consequences of prenatal caring. This process was supported by the use of ATLAS.ti software, version 22.

3. Reduction: The codes were reviewed and discussed by the research team, then grouped according to antecedents, attributes, and consequences of the concept of prenatal caring.

Three categories emerged from the analysis of the interviews with study participants. The first category, "The nurse's role in low-risk prenatal caring," describes the nurses' actions in prenatal caring as expressed by pregnant women. This category includes a subcategory of analysis titled "Low-risk prenatal nursing consultation: A space for interaction between the nurse and the pregnant woman." 

The second category, titled "Low-risk prenatal nursing consultation: A space for interaction between the nurse, the pregnant woman, and her family," describes the characteristics and attributes that distinguish nursing professionals from other members of the healthcare team involved in the prenatal care of low-risk pregnant women. This category comprises two subcategories of analysis: "Characteristics and attributes of prenatal caring" and "Values of nursing professionals implicit in prenatal caring." The third category, titled "Prenatal caring nursing: A commitment to the pregnant woman and her unborn child," describes the outcomes of the interaction between nurses and the pregnant woman, as well as the contributions of nursing professionals to the well-being of the mother-child dyad during the prenatal period. 

**The nursing role in low-risk prenatal caring**

The role of nursing in low-risk prenatal caring, as expressed by pregnant women and nurses, encompasses several routine activities performed during prenatal consultations. These activities include assessment of maternal anthropometry (such as weight, height, uterine height measurements), monitoring of weight gain during pregnancy, blood pressure monitoring, ordering of screening tests and/or laboratory analysis, prescription of micronutrients, and referral of pregnant women to specialized consultations as needed. All these technical and operational elements are recognized by pregnant women as characteristic of the caring provided by nursing professionals, as illustrated by the following statement:

*“At the prenatal visit, the nurse weighed me, checked my blood pressure, measured my belly to see how much it was growing, gave me vitamins, ordered some tests, and referred me to other professionals...” (Interview G4) *

Undoubtedly, a significant portion of low-risk prenatal care is protocol-driven, and prenatal nursing care is often perceived as a mechanical task constrained by limited consultation time.

*“As a prenatal nurse, I make sure that everything I do follows the standards, guidelines and protocols...” (Interview E5) *

In pursuing comprehensive prenatal care, nurses go beyond the technical and operational aspects of the prenatal checkup consultation. The perspective of prenatal caring transcends the physiological aspects of pregnancy, viewing the pregnant woman as a unique individual shaped by her interactions with her environment.

*"...we don't just assess the physical part, but also the emotional and family aspects, because all of these influence how the pregnancy develops..." (Interview E4) *

Prenatal education is another key component of the nursing role in prenatal care. Both nurses and pregnant women recognize this activity as a fundamental aspect of pregnant women's care during the prenatal period. Typically, prenatal education focuses on identifying warning signs and symptoms during pregnancy, addressing unresolved questions and concerns from previous consultations, and ensuring that the information provided during the visit is fully understood.

*"I talk to them about the care during pregnancy, the warning signs to watch out for. I also clear up any doubts they bring from other consultations, like nutrition or gynecology." (Interview E5) *

*"The nurse explains things in a way I can understand, not so technical. That’s really important because, obviously, there are a lot of words you professionals use that we don’t know." (Interview G4) *

Another noteworthy aspect of the prenatal nurse's role is the follow-up with the pregnant woman throughout the prenatal period. This activity entails significant professional responsibility and continues to face important limitations; nonetheless, pregnant women view it positively.

*"...we keep following up with her, because there are pregnant women that—even if I’m not the one seeing them—I still know what their situation is." (Interview E1)*

*"They were very attentive—they called me for appointments and were always in touch with me." (Interview G5)*

**Low-risk prenatal nursing consultation: a space for interaction between the nurse, the pregnant woman, and her family**

Low-risk prenatal nursing consultations serve as a space for interaction between the nurse, the pregnant woman, and her family. They take place in a quiet place, allowing the pregnant woman to feel at ease with a nurse who possesses the knowledge necessary to provide safe, responsible, and warm prenatal caring. This type of consultation fosters the sharing of experiences related to the pregnancy process and is distinguished by the love and genuine concern that nursing professionals demonstrate toward the woman.

*"...the prenatal consultation should happen in a quiet, calm space where pregnant women feel comfortable—a place where they can share their experiences." (Interview E4) *

*"...not just anyone can provide prenatal caring—it’s not just about having the knowledge, but also about having a real love for caring for pregnant women." (Interview E2) *

*"...the nurse always brings that warmth and closeness, and gives expectant mothers the chance to feel comfortable and at ease." (Interview G2) *

*"...I felt like my relationship with the nurse was like being with a good friend—or even like a mother—because they were always concerned about me and constantly looking out for me." (Interview G5) *

*"...it's about building trust with pregnant women, so they feel well cared for and can share the best information with us—so we can provide prenatal caring that truly promotes maternal and perinatal well-being." (Interview E1) *

*"A characteristic part of the prenatal consultation is building a bond of trust with the pregnant woman. That happens when I introduce myself as a nurse, make her feel assured, ask about her background, show genuine concern for what she’s going through—just by being honest and being myself." (Interview E1) *

*"Knowledge, assurance, and responsibility are three characteristics that every nurse providing prenatal caring should have." (Interview E4) *

**Characteristics and attributes of prenatal caring**

Prenatal caring possesses specific characteristics and attributes that make the encounter between the participants unique. The empathy demonstrated by nursing professionals is seen as a gateway to prenatal caring. This attitude allows pregnant women to feel welcomed by nurses who engage in active listening, express sensitivity^3^, and show genuine interest in supporting them throughout the gestational process. Women attending low-risk prenatal nursing consultations value the interest nurses show in the progression of their pregnancies. They perceive nurses as more approachable, attentive, and committed to their role (Table 4). 

**Table 4:** Characteristics and attributes of prenatal caring

Empathy	"The characteristics that stand out in nursing are empathy, respect, responsibility—they’re always there when you need them..."(Interview G3) "The way she cares for me makes me feel supported—I don’t feel rejected, scolded, or anything like that." (Interview G1)
Welcoming attitude	"The nurse was very attentive—she answered all my questions, and honestly, I felt really good during my checkups." (Interview G1) "Whenever I came in for my prenatal consultation with the nurse, I could always see her willingness to see me—I always felt welcomed by her." (Interview G5)
Concern	"The head nurse cared about whether I showed up, had my checkups, and got my tests done—he followed up with me like he was genuinely concerned about me." (Interview G2)
Active listening	"The nurse made me feel good because she listened to me. I would tell her everything that was going on, and she would give me personal advice. You could say they were really involved and understood me." (Interview G5) "I told her little things that happened at home—things that might seem silly—but she always listened and paid attention to everything I said." (Interview G5)
Sensibility	"I think one of the characteristics that prenatal caring should have is the nurse's sensitivity and humanized care..." (Interview G2)
Assurance	"Here, the nurse explained things to me in more detail, and because of that, I felt assured going home." (Interview G2)
Confidence	"He gave me confidence, opened doors for me—it was something different." (Interview G2)

**Implicit values of nursing professionals in prenatal caring**

One characteristic of nursing professionals who provide prenatal caring is the personal and professional value system implicit in it. These values—closely linked to the identity and practice of being a nurse—serve as key facilitators of caring and promote adherence to prenatal care. Among the values described by the study participant, respect, responsibility, and kindness stand out. These three values underpin the actions of nursing professionals, shaping and harmonizing their interactions with the pregnant woman and her family throughout the prenatal period (Table 5).

**Table 5:** Implicit values of nursing professionals in prenatal caring

Respect	"I always found the head nurse to be respectful and kind. I'm not saying the doctor wasn't, but with the nurse, it was much more noticeable." (Interview G2).
Responsibility	"As a prenatal nurse, I feel a strong sense of responsibility—that everything goes well, that the pregnancy reaches full term, that the pregnant women and their families feel satisfied, and that everything I do follows the standards, guidelines, and protocols." (Interview E5)
Kindness	"Whenever I came in for my prenatal consultation with the nurse, I could always see her willingness to care for me, her kindness—that gave me confidence." (Interview G5)

**Prenatal caring nursing: a commitment to the woman and her unborn child**

Prenatal nursing care contributes to reducing maternal and perinatal mortality through the early detection of pregnancy-related complications by nursing professionals. However, prenatal caring nursing transcends the systematic aspects of prenatal care. Prenatal caring nursing fosters the pregnant woman’s commitment to both self-care and the care of her unborn child, allowing the experience to unfold in a positive way, integrating physical and emotional preparation for childbirth and motherhood.

*"As nurses, we contribute to prenatal care by detecting maternal risks in a timely manner to help prevent maternal and perinatal deaths." (Interview E3)*

In the analytical phase, the findings from the theoretical and fieldwork phases were compared to generate a concise definition grounded in both the literature and the participants' perspectives. As a result, the final definition was supported by both theoretical and empirical data. 

**Concept antecedents**

Low-risk prenatal nursing care is grounded in scientific evidence from nursing^3^,^32^,^43^ and related disciplines^26^,^31^,^32^ and is notably influenced by the biomedical model^28^,^30^,^38^,^43^,^45^. It is periodic, sequential, comprehensive, integrated, and continuous^26^,^29^,^45^. The provision of such care requires specific professional competencies^26^,^29^,^30^,^40^. Prenatal care includes the physical and psychosocial assessment of the pregnant woman ^26^,^27^,^29^,^30^,^35^, the implementation of protocolized activities during low-risk prenatal consultation^26^,^27^,^29^,^30^,^43^, the delivery of prenatal counseling and education^29^,^35^,^40^,^46^, follow-up, and care management throughout the prenatal period^26^,^29^,^30^,^43^,^45^. This care is also humanized and centered on the woman, her unborn child, her partner, or her family ^26^,^31^,^32^,^35^,^36^,^45^. It arises from the interactive process between nursing professionals, the pregnant woman, and her family during the prenatal period ^27^,^28^,^30^,^35^,^36^. 

**Attributes of the concept**

Nurses who provide prenatal caring demonstrate sensitivity to issues related to women's sexual and reproductive health, particularly during the gestational and prenatal periods^27^,^32^,^35^,^36^,^38^. Providing prenatal caring requires understanding the fears, anxieties, and uncertainties experienced by pregnant women and their families^26^,^27^,^35^,^36^, as well as recognizing conditions of vulnerability that go beyond the physiological aspects of pregnancy^3^,^26^,^31^,^32^,^39^. Prenatal caring fosters bonds that generate a sense of commitment to the care of the pregnant woman, her unborn child, her partner, and her family^26^,^29^,^30^,^37^,^40^. Within this interactive process, the nurse's genuine concern for the well-being of the mother-child dyad^26^,^29^,^37^,^40^ becomes evident and is expressed through kindness, respect, and responsibility^26^,^29^,^30^,^37^,^40^. Prenatal caring is marked by empathy^27^,^32^,^35^,^36^,^38^, attentiveness, and active listening. It involves a welcoming attitude, assurance, and trust, allowing the pregnant woman and her family to feel supported ^3^,^26^,^31^,^32^,^39^. 

**Concept consequences**

From the perspective of nursing professionals, prenatal caring promotes the pregnant woman's commitment to both self-care and the care of her unborn child. This approach enables the pregnancy experience to unfold in a positive way by supporting the woman's physical and psychological preparation for childbirth and motherhood. The quality of care fosters satisfaction, which in turn encourages adherence to low-risk prenatal nursing care. Ultimately, these outcomes contribute to the reduction of maternal and perinatal mortality^29^,^31^,^32^,^35^,^40^. 

**Final definition**

Prenatal caring is the interaction process between the pregnant woman, her unborn child, her family, and nursing professionals during the prenatal period. This process is grounded in values such as kindness, respect, and responsibility, and it fosters commitment to the care of the pregnant woman. Prenatal caring involves empathy, attentiveness to the needs of the woman and her family, a welcoming attitude, the ability to inspire assurance and trust, and transcending the technical and physiological aspects of pregnancy.

## Discussion

The results of this research are presented through the analysis of the antecedents, attributes, and consequences of the concept of prenatal caring as perceived by a group of pregnant women and nursing professionals responsible for low-risk prenatal consultations. 

In this research, it became evident that the terms "prenatal care" and "prenatal caring" are often used interchangeably by healthcare providers. However, analyzing the participants' statements suggests a clear conceptual distinction between the two. 

In practice, the operationalization of prenatal caring tends to be ambiguous, as the terms "taking care" and "caring for" are often treated as synonymous and used interchangeably. From a pragmatic perspective, however, the use of the concept of prenatal caring extends beyond the execution of technical and procedural aspects of prenatal care. In this regard, Hernández and Vásquez^35^ emphasize that prenatal care should not be limited to technical tasks associated with pregnant women' care but should transcend the operational issues of this process. 

The results from the literature review are contrasted and supported by the voices of nursing professionals and pregnant women, shaping prenatal caring as a theoretical construct that relates to the interactional process that unfolds between the woman, her partner, her family, and the nursing professionals during pregnancy. According to Ruiz and Muñoz^10^, this encounter constitutes a particular form of intersubjective relationship in which the involved parties (health professionals, the pregnant woman, and her family) share experiences that reveal genuine care needs, and it goes beyond the performance of activities, procedures, and interventions aimed at the early detection of pregnancy-related disorders. 

Prenatal caring is materialized in the prenatal checkup and follow-up nursing consultations for women with low-risk pregnancies. These consultations provide a space in which the nurse, the pregnant woman, her unborn child, her partner, and her family interact in an environment that fosters comfort and ease to share their experiences regarding prenatal caring^27^,^31^,^32^,^35^,^40^. 

The pregnant women who attended the low-risk prenatal nursing consultations appreciated the interest shown by nurses throughout their gestational process and perceived them as more approachable, attentive, and committed to their work. In this regard, Hernández and Vásquez^35^ emphasize that nurses' interest and sense of responsibility toward the well-being of the pregnant woman and her unborn child foster feelings of assurance and trust. These sentiments, in turn, motivate women to continue attending prenatal checkups. 

Prenatal care is materialized with prenatal checkup consultations, which can be conducted by nursing professionals. In operational terms, the prenatal checkup consultation refers to a series of scheduled contacts, interviews, or visits between a pregnant woman and the healthcare professionals responsible for prenatal care. Its purpose is to monitor the progression of the pregnancy and ensure adequate preparation for childbirth and parenting^3^,^27^,^28^,^31^,^32^,^40^. 

In the context of healthcare in Colombia, the participation of nursing professionals in prenatal care is highly diverse. Some health institutions incorporate nurses into the Integrated Healthcare Pathway for Maternal and Perinatal Population^49^, recognizing their potential to offer a comprehensive approach to the care of pregnant women and their families. Other institutions restrict nurses' involvement in the care of women with low-risk pregnancies, a practice that varies across regions and levels of care within the country. In many cases, pregnant women classified as high obstetric risk from the outset do not have the opportunity to be assessed by nursing professionals, as this classification often leads to their automatic exclusion from nursing-led care. 

This study highlights the role of nursing professionals in prenatal counseling and education, a role that has earned them significant recognition as integral members of the prenatal care team providing perinatal care. Studies such as Bohren's^2^ indicate the importance of counseling for both the pregnant woman and her companion within prenatal caring. These moments of interaction foster relationships grounded in trust, which, in turn, promote the pregnant woman’s autonomy in caring for herself and her child. Therefore, a relationship based on mutual exchange and reciprocity is necessary. 

This study identified the attributes of the concept of low-risk prenatal caring nursing in the context of maternal-perinatal care. Providing prenatal caring from a nursing perspective involves understanding the fears, anxieties, and uncertainties experienced by the pregnant woman, her partner, and her family. It requires recognizing conditions of vulnerability transcending the physiological aspects of pregnancy^3^,^26^-^28^,^31^,^32^,^35^,^36^. Likewise, a study conducted in Brazil^39^ affirms that a nurse who actively listens to a woman’s doubts and fears, allows her to express herself, and identifies her needs is able to offer immediate caring that provides reassurance and conveys a sense of trust during the consultation. 

Prenatal caring creates bonds that generate a sense of commitment to the caring of the pregnant woman, her unborn child, her partner, and her family^26^,^31^,^34^,^35^. Within this interactional process, genuine concern for the well-being of the mother-child dyad becomes evident, grounded in kindness^27^,^35^,^36^,^38^, respect^26^,^32^,^35^,^36^,^38^, and responsibility^26^,^29^,^30^,^37^,^40^. This form of caring involves empathy, attentiveness through active listening^27^,^28^,^35^,^36^, a welcoming attitude^26^,^27^,^35^,^36^, assurance, and trust^27^,^32^,^35^,^36^,^38^, allowing the pregnant woman, her partner, and her family to feel supported. In line with this, Amorim et al.^29^ emphasize that prenatal care activities should be directed to the pregnant woman and carried out with the highest level of dedication, attentiveness, and demonstrated commitment. It should involve welcoming the pregnant woman and her companion to promote optimal maternal well-being and ensure the best possible health for the mother and child. 

A situational analysis of nursing professionals' participation in low-risk prenatal consultation in the city of Monteria shows how they assume responsibility for providing prenatal care. However, their participation is largely concentrated on operational tasks and care management, resulting in uncoordinated, fragmented, and solution-oriented care. 

With the implementation of the Integrated Healthcare Pathway for Maternal and Perinatal Population, nursing professionals have assumed an important leadership role in prenatal care. However, their role in this process has often been limited to addressing the operational aspects of prenatal care. Their role has been largely confined to attending to the pregnant woman and her unborn child, with their actions primarily focused on the admission process into the Integrated Healthcare Pathway for Maternal and Perinatal Population. 

Nursing professionals have gradually lost space for interaction with pregnant women and their families. Consultation times are increasingly limited, and responsibilities, once led by nurses, have been delegated to other members of the healthcare team. For this reason, it is important that nursing professionals reclaim and strengthen their roles, reasserting their leadership in prenatal care processes. Doing so will enable comprehensive, integrated, and continuous prenatal care in which the boundaries and scope of nursing practice are clearly recognized. 

The Colombian Association of Nursing Faculties (ACOFAEN in Spanish) outlines the competencies of nursing professionals in prenatal care, emphasizing that nurses are qualified to provide high-quality care, lead care processes, and collaborate with the interdisciplinary team that provides prenatal care. Additionally, the National Nursing Human Resource Policy reaffirms the professional competencies of nurses in Colombia to lead healthcare processes across the areas of healthcare, education, research, and management. 

The conceptual proposal derived from this study represents a contribution to nursing practice, as it helps clarify nursing professionals' boundaries and spheres of action. It invites them to review and reflect on the care and nursing role supporting the pregnant woman, her unborn child, her partner, and her family throughout the prenatal or antenatal period. 

## Conclusions

The concept analysis method developed by Schwartz-Barcott and Kim has enhanced the understanding of prenatal caring and broadened its application within the maternal–perinatal context. This analysis has contributed to describing the attributes of caring nursing for pregnant women at low obstetric risk, offering a novel theoretical perspective to inform both practice and research. The way prenatal care is defined in nursing practice depends on the biomedical model and obstetric risk classification, which constrains the scope of nursing involvement in prenatal care. There is a need to adopt other types of care approaches to ensure the practice of prenatal care and a political commitment to integrate contributions of the nursing discipline within this specific field of knowledge. 

In nursing, prenatal caring involves understanding the emotional needs and conditions of vulnerability experienced by the pregnant woman and her family while fostering bonds of commitment to their care. The positive prenatal care experience contributes to increased adherence, reduced maternal and perinatal mortality, and comprehensive preparation for childbirth and puerperium. It calls for a vindication of prenatal caring nursing as a space for interaction and learning centered on the pregnant woman, her unborn child, her partner, and the family. 

## References

[1] World Health Organization (2016). WHO recommendations on antenatal care for a positive pregnancy experience. WHO. [Internet].

[2] Bohren  MA, Hofmeyr  GJ, Sakala  C, Fukuzawa  RK, Cuthbert  A (2017). Continuous support for women during childbirth. Cochrane Database Syst Rev.

[3] Taheri  M, Takian  A, Taghizadeh  Z, Jafari  N, Sarafraz  N (2018). Creating a positive perception of childbirth experience: systematic review and meta-analysis of prenatal and intrapartum interventions. Reprod Health.

[4] Heberlein  EC, Picklesimer  AH, Billings  DL, Covington-Kolb  S, Farber  N, Frongillo  EA (2016). The comparative effects of group prenatal care on psychosocial outcomes. Arch Womens Ment Health.

[5] Fiscella  K (1995). Does prenatal care improve birth outcomes? A critical review. Obstet Gynecol.

[6] Côté-Arsenault  D,  Hubbard  LJ (2019). Improving Perinatal Care Through Theory Application. MCN Am J Matern Child Nurs.

[7] Morse  JM, Mitcham  C, Hupcey  JE, Tasón  MC (1996). Criteria for concept evaluation. J Adv Nurs.

[8] Lassi  ZS, Middleton  PF, Crowther  C, Bhutta  ZA (2015). Interventions to Improve Neonatal Health and Later Survival: An Overview of Systematic Reviews. EBioMedicine.

[9] Nwabueze  CO, Okeke  CC, Nwevo  CO, Nwodo  LA, Nwekpa  WC,  Nwaiwu  PI (2023). Assessing Focused Antenatal Care Awareness and Utilization Among Pregnant Women in Enugu State, Nigeria: A Cross-Sectional Survey. Cureus.

[10] Ruiz  C, Muñoz  L (2006). Cuidado de enfermería materno perinatal en su rol asistencial, gerencial, docente e investigativo.

[11] Fondo de Población de las Naciones Unidas (UNFPA), Ministerio de Salud y Protección Social (2013). Protocolos para la atención de enfermería a la salud sexual y reproductiva de la mujer.

[12] Yuen  WS, Lo  HC, Wong  WN, Ngai  FW (2022). The effectiveness of psychoeducation interventions on prenatal attachment: A systematic review. Midwifery.

[13] Rodgers  B (2000). Philosophical foundations of concept development..

[14] Rodgers  Beth L, Kathleen  Astin Knafl (2000). Concept development in nursing: foundations, techniques, and applications. An expansion and elaboration of the hybrid model of concept development.

[15] Hardy  ME (1978). Perspectives on nursing theory. ANS Adv Nurs Sci.

[16] Walker  L, Avant  K (2010). Strategies for theory construction in nursing.

[17] Rodgers  BL,  Jacelon  CS, Knafl  KA (2018). Concept Analysis and the Advance of Nursing Knowledge: State of the Science. J Nurs Scholarsh.

[18] Fawcett  J, Garity  J (2008). Evaluating research for evidence-based nursing practice. Fa Davis. [Internet].

[19] Morse  JM (2016). Concepts in context. En: Analyzing and conceptualizing the theoretical foundations of nursing.

[20] Schwartz-Barcott  D, Kim  HS (2000). An expansion and elaboration of the hybrid model of concept development.

[21] Moser  A,  Korstjens  I (2017). Series: Practical guidance to qualitative research. part 1: Introduction. Eur J Gen Pract.

[22] Castillo  ME,  Vásquez  ML (2003). El rigor metodológico en la investigación cualitativa. Colombia Médica.

[23] Otzen  T, Manterola  C (2017). Técnicas de Muestreo sobre una Población a Estudio. Int J Morphol.

[24] Bula-Romero  JA, Conde-Flórez  AA, González-Lara  AM, Sánchez-Caraballo  ÁA, Jiménez-Hernández  GE (2025). Articles analyzing the concept of nursing care. Mendeley Data, V1.

[25] Ministerio de Salud y Protección Social Resolución número 3280 de 2018, por medio de la cual se adoptan los lineamientos técnicos y operativos de la Ruta Integral de Atención para la Promoción y Mantenimiento de la Salud y la Ruta Integral de Atención en Salud para la Población Materno Perinatal y se establecen las directrices para su operación.

[26] Simão  AMS, Santos  JLG dos, Erdmann  AL, Mello  ALSF de, Backes  MTS, Magalhães  ALP (2019). Management of prenatal nursing care at a Health Center in Angola. Rev Bras Enferm.

[27] Gomes  CB de A, Dias  R da S, Silva  WGB, Pacheco  MAB, Sousa  FGM de, Loyola  CMD (2019). Prenatal nursing consultation: Narratives of pregnant women and nurses. Texto e Context Enferm.

[28] Soares  CS, dos Santos  NO, Diaz  CMG, Pereira  SB,  Bär  KA, Backes  DS (2021). Nursing consultation in prenatal care from the perspective of postpartum women: an exploratory-descriptive study. Online Brazilian J Nurs.

[29] Amorim  TS,  Backes  MTS, Carvalho  KM de, Santos  EKA dos, Dorosz  PAE, Backes  DS (2022). Gestão do cuidado de Enfermagem para a qualidade da assistência pré-natal na Atenção Primária à Saúde. Esc Anna Nery.

[30] Sehnem  GD, de Saldanha  LS, Arboit  J, Ribeiro  AC,  de Paula  FM (2020). Prenatal consultation in primary health care: Weaknesses and strengths of Brazilian nurses’ performance. Rev Enferm Ref.

[31] Moreno Mojica  CM, Mesa Chaparro  NP, Pérez Cipagauta  Z, Vargas Fonseca  DP (2015). Convertirse en madre durante la adolescencia: activación del rol materno en el control prenatal. Rev Cuid.

[32] Jacob  T de NO, Rodrigues  DP, Alves  VH,  Ferreira  E da S, Carneiro  MS, Penna  LHG (2021). The perception of woman-centered care by nurse midwives in a normal birth center.. Esc Anna Nery.

[33] Lima  KSV, Carvalho  MM de B, Lima  TMC, Alencar  D de C,  de Sousa  AR, Pereira  Á (2021). Father’s participation in prenatal care and chilbirth: contributions of nurses’ interventions. Nurs Res Educ.

[34] Cáceres-Manrique  FM, Molina-Marín  G, Ruiz-Rodríguez  M (2014). Maternidade: Um processo com diferentes nuances e construção de vínculos. Aquichan.

[35] Hernández Betancur  AM, Vásquez Truisi  ML (2015). Committed nursing care: Engine satisfaction of pregnant women during prenatal care. Univ y Salud.

[36] López Hidalgo  R, Hernández Segura  GA, Gallegos Torres  RM (2018). Perception of pregnant women of the interaction with the nursing staff in the prenatal control, in the city of San Cristobal de las Casas. Chiapas, Mexico. Horiz enferm.

[37] Guerra  N, Reina  R, Cárdenas  MH, Sanmiguel  F (2019). Significado del cuidado de la gestante desde la experiencia de la enfermera. Rev Científica Ágora.

[38] Ferrer de Oliveira  LL, Soares Figueredo Trezza  M, Pereira dos Santos  A, Cavalcante de Melo  G, Torres de Lima Sanches  ME, Tenorio Ribeiro Pinto  LM (2017). As vivências de conforto e desconforto da mulher durante o trabalho de parto e parto. Rev enferm UERJ.

[39] Jiménes López  E, Ponce Gómez  G (2019). Caring for pregnancy: The case of Tseltal midwives in Chiapas, Mexico. Cult los Cuid.

[40] Benedet  DCF, Wall  ML, Lacerda  MR, Machado  AV de MB, Borges  R, Zômpero  JFJ (2021). Strengthening nurses in prenatal care through reflection-action. Rev Gauch Enferm.

[41] Castiblanco-Montañez  RA, Berruecos-Prada  DC, Calderón-Rivas  EM, Guayacundo-Aldana  MJ,  Mancera-García  RM, Rodríguez-Ramírez  KS (2021). Enfermera-matrona: beneficios, competencias e intervenciones. Rev. cienc. cuidad..

[42] Guarnizo-Tole  M, Olmedillas  H, Vicente-Rodríguez  G (2018). Evidencia del aporte proporcionado desde el cuidado de enfermería a la salud materna. Rev Cuba Salud Pública.

[43] Garcia  ESGF, Bonelli  MCP, Oliveira  AN, Clapis  MJ, Leite  EPRC (2018). The Nursing Care Actions Toward the Pregnant women: Challenging the Primary Health Care. Rev Pesqui Cuid é Fundam Online.

[44] Reyes Bravo  DM, Muñoz de Rodriguez  L (2019). Valoración del cuidado de enfermería por parte de adolescentes gestantes antes de una intervención en atención prenatal y después de esta. Investig en Enfermería Imagen y Desarro.

[45] Serruya  SJ, Cecatti  JG,  Lago  T di G do (2004). O Programa de Humanização no Pré-natal e Nascimento do Ministério da Saúde no Brasil: resultados iniciais. Cad Saude Publica.

[46] Chaves  IS, Rodrigues  IDCV, Freitas  CKAC, Barreiro  M do SC (2020). Pre-natal consultation of nursing: satisfaction of pregnant women. Rev Pesqui Cuid é Fundam Online.

[47] Corominas  J (1954-1997). Diccionario Crítico Etimológico de la Lengua Castellana: Etimología de Cuidado.

[48] Corominas  J (1954-1997). Diccionario Crítico Etimológico de la Lengua Castellana: Etimología de Prenatal.

[49] Ministerio de Salud y Protección Social – Colciencias (2013). Guía de práctica clínica para la prevención, detección temprana y tratamiento de las complicaciones del embarazo, parto o puerperio.

